# Relative Value of Turtle Tuberculin in the Treatment of Tuberculosis*From the New York Medical Journal of October 25th.

**Published:** 1913-12

**Authors:** Edward E. Myers

**Affiliations:** 418 Central Park West, New York City


### Relative Value of Turtle Tuberculin in the Treatment of Tuberculosis.*

(Second Paper.)

BY DR. EDWARD E. MYERS, 418 CENTRAL PARK WEST, NEW YORK CITY.

All the precautionary measures devised by science in late years for checking the advance of tuberculosis, including sanitation, out-of doors living, hygienic legislation and the like have failed to arrest the development of the disease. The annual death toll of tuberculosis reaches the awful figure of two hundred thousand in this country alone, and throughout the world this disease claims one human life every two minutes and a half.

Robert Koch's revolutionizing discovery of the tubercle bacilli has put science upon the right track and since then great progress has been made. Thanks to the research work by Professor Piorkowski of Berlin, a specific curative and immunizing agent—his turtle tuberculin—as indicated by the collective experience of Dr. Beattie and Dr. Myers bids fair to herald a new era in the specific treatment of consumption.

Piorkowski believes that an intravenous injection of his turtle tuberculin combines with the receptors of Koch's side-chain theory and forms an antitoxin similar to Jenner's vaccine for small-pox, and far superior in curative properties to that formed by injections of living human tubercle bacilli which admittedly attained a certain result, albeit an inadequate one.

In response to many inquiries since the appearance of the first article on Piorkowski's turtle tuberculin in the *New York Medical Journal* of September 13, 1913, on the "Relative Value of Turtle Tuberculin in the Treatment of Tuberculosis," the following specific results may be recorded in four of the cases treated.

Case XV. An inspector in the Custom House Service of the

*From the New York Medical Journal of October 25th.

United States Government, 32 years of age, diagnosticated by several competent physicians as presenting all the physical signs and symptoms of tuberculosis of the lungs, having been ill since about September, 1909, and having fallen off from 175 pounds to 105 pounds, and becoming too weak to hold his knife and fork in his hand responded in less than four months to the Piorkowski turtle tuberculin treatment increasing in weight to 159½ pounds, losing his cough, his pains in the chest and other symptoms, and repeated examinations have failed to discover a single symptom of the disease from which he had been suffering for more than three years. Repeated bacteriological examinations by the New York Board of Health have not disclosed any trace of the presence of tuberculosis. Therefore this case may be considered a specific cure.

Case XXI. A white girl, aged 7 years, suffering from tuberculosis of the knee joint for over two years, responded to the Piorkowski treatment in a period of less than four months, to the extent of increasing the motion of the affected joint 50 per cent. The treatment resulted in great general improvement, including the reduction of one-half inch of the swelling of the knee joint and a gain of over six pounds in weight.

Case XXVII. A white girl, aged nineteen years, suffering from tubercular glands of the neck since 1909, responded to less than four months' treatment with Piorkowski's turtle tuberculin by a gain of eight pounds, an increase of appetite and a marked improvement in general condition. Only a few small glands remained with no discharging sinus, where formerly had been a large irregular mass of glands with a discharging sinus.

Case XLIV. This case of a man aged 44 years is cited more particularly to bring out laryngeal tuberculosis than a condition of the lungs. The patient had been hoarse for some months and treatment extending to a little over one month gradually eliminated the hoarseness, signs were practically absent in the larynx and there was improvement in the cough, expectoration and color of sputum. The patient retained his weight, although the tuberculosis condition was complicated with a severe form of diabetes. One month of treatment resulted in an improvement greater than that attained during the previous eight months under other forms of treatment.

---

If vomiting after a laparotomy persist in spite of treatment, especially in cachetic individuals, inspect the wound. Occasionally the cause is prolapse of abdominal contents through the opened incision.—*American Journal of Surgery.*

